# Supplementary material for: Canadian status of “not acceptable” drugs as evaluated by Prescrire: A cohort study
Source: PLoS One. 2024 Aug 1;19(8):e0308118. doi: 10.1371/journal.pone.0308118 (PMC11293654; doi:10.1371/journal.pone.0308118)
Supplement: Attachment — Submitted filename: 20240524-PONE-D-24-11119_review comments.docx [file pone.0308118.s002.docx]

**Title: Status of “not acceptable” drug-indications in Canada: a cohort study**

**Journal: PLOS ONE**

**Manuscript number: PONE-D-24-11119**

Reviewers’ Comments:

Dear Author, dear Editors,

This study aims to assess the status of drugs in Canada that have been rated as ‘not acceptable’ by the French drug bulletin Prescrire. In general, the manuscript adheres to most of PLOS ONE’s publication criteria. However, especially the analyses performed and the reporting and contextualization of the results require some more depth. Therefore, I deem several major revisions necessary before I can recommend it for publication. In that light, I have several questions and suggestions for the author to clarify the study and improve the analyses and the presentation of the results.

**Major**

- Although highly specialised readers in this field may instantly appreciate the choice of using Prescrire’s ratings of drugs, the manuscript lacks a clear rationale why this study aims to use data from Prescrire for the Canadian context. The introduction scarcely mentions what Prescrire is.
- Similarly, the Limitations section (p.11) states that there is no independent confirmation of Prescrire’s ratings. However, several studies have compared therapeutic value as assessed by Prescrire with that assessed by (European) HTA agencies. Their findings add important context and shed light on the generalisability of Prescrire’s ratings. I thus recommend the author to clarify the choice of using Prescrire to make the study more accessible and understandable for non-specialists as well as to readers who are familiar with assessing the therapeutic value of new drugs.
- While a lot of studies have evaluated therapeutic value of new drugs as assessed by (different) regulatory authorities and differences between them and HTA organisations, the manuscript lacks grounding in and connection of its findings and rationale to these studies and discussions. I believe this would add to the manuscript’s relevance and rationale when these discussions are referred to and reflected upon both in the introduction and the discussion section.
- The study’s Methods are in principle fit for answering the presented research question, yet some explanation would help to understand why and which steps were undertaken. For instance, it is yet unclear why data about the completion of the post-authorisation conditions were collected for drugs with a NOC/c approval and why the safety advisories were collected. Moreover, providing more context and detail to the data analysis section would help in understanding why steps are taken.
- In relation, the completion status and safety advisories not necessarily only lead to drug withdrawals, although the last sentence of the first Results paragraph (p.8, first sentence) and the second-last Results paragraph do suggest this. This should be further explained.
- In general, the Results section requires restructuring and additional headings to better guide the reader through the study’s findings and their relevance.
- To support and make the study’s findings more intelligible, the Results section would benefit from adding a table and/or figure (such as bar or pie charts) in which the findings are presented. Similarly, a typical ‘Table 1’ would provide required information on the drugs and indications included in the study (n=62/70). Figure 1 would benefit from a clearer visual distinction between the included and excluded drugs. Of note, the ‘safety advisories issues’ now suggest exclusion of these drugs from the cohort. Moreover, clarifying how the results and percentages relate to the different ‘buckets’ of medicines would be helpful for readers.

**Minor**

- I recommend to revise the title and abstract to clarify that the ‘not acceptable’ rating is as considered by Prescrire. Now, they suggest that the study assessed drugs that were refused for marketing authorization by Health Canada. This should also be clarified throughout the manuscript.
- As mentioned in the Limitations section, the data collection has been performed only by the author himself. Given the vast number of bulletins that needed to be checked manually, I would like to emphasise the importance to validate at least a sample by a second researcher.
- I must admit that I could not locate the difference in appraisals between Prescrire and Health Canada as discussed in the second sentence of the abstract, in the Results section. At least, it was not explicitly discussed.
- The last paragraph of the Results (“Twenty-seven of the (…) in favour of the EMA/ANSM” (p.9) may be moved more forward, for a more logical flow of the Results section.
- The manuscript’s intelligibility would profoundly benefit from more detail and explanation throughout the manuscript, including:
- Explain the relation between the therapeutic evaluations by the PMPRD and the study’s objective (as for instance mentioned in abstract)
- How are ‘drug-indications’ defined?
- The methods section describing the data analysis is rather concise, yet, more clarification would help the reader to understand how this relates to the results presented
- Introduction:
  - First paragraph, the ‘EMA’s decentralized procedure’ is not governed by EMA nor does it allow approval in a single market per definition
  - Clarify the objective: is the aim to assess only whether they were approved? Should be aligned.
- Methods/Results: why is the formulation relevant for the objective? If necessary, please explain.
- The last sentence of second paragraph of the Discussion on p10 is a relevant observation. Could such differences in evaluation outcomes between PMPRB and Prescrire be due to differences in availability of alternative treatments in Canada vs. France/the EU?
- Also, given the highly related topic of ref 12, it should also be discussed in the introduction.
- The Data Availability Statement states that all data are available in supplementary, however, is the data from Prescrire approved to be made available?
- Finally, the manuscript would be improved in clarity and coherence by specifying the actors in several sentences. For instance, in the sentences below Figure 1 on page 8-9: who rated the ‘slight/no improvement’; and who did the ‘therapeutic evaluations’; which review process. Also in the second sentence of the Discussion: who performed the therapeutic ratings. Etc. etc.
