# Supplementary material for: Canadian status of “not acceptable” drugs as evaluated by Prescrire: A cohort study
Source: PLoS One. 2024 Aug 1;19(8):e0308118. doi: 10.1371/journal.pone.0308118 (PMC11293654; doi:10.1371/journal.pone.0308118)
Supplement: Attachment — Submitted filename: Response to editors and reviewers.docx [file pone.0308118.s003.docx]

**Response to editors**

1. When submitting your revision, we need you to address these additional requirements.

Please ensure that your manuscript meets PLOS ONE's style requirements, including those for file naming. The PLOS ONE style templates can be found at  <https://journals.plos.org/plosone/s/file?id=wjVg/PLOSOne_formatting_sample_main_body.pdf> and  <https://journals.plos.org/plosone/s/file?id=ba62/PLOSOne_formatting_sample_title_authors_affiliations.pdf>

*I have carefully read PLOS ONE’s style requirements and can confirm that my manuscript meets those requirements.*

2. Thank you for stating the following in the Competing Interests section:  Between 2020-2024, Joel Lexchin received payments for writing a brief on the role of promotion in generating prescriptions for a legal firm, for being on a panel about pharmacare and for co-writing an article for a peer-reviewed medical journal. He is a member of the Board of Canadian Doctors for Medicare. He receives royalties from University of Toronto Press and James Lorimer & Co. Ltd. for books he has written. He is participating in research funded by the Canadian Institutes of Health Research.     We note that one or more of the authors are employed by a commercial company.

*Please read my COI statement carefully. It does NOT say that I am or have been employed by a commercial entity. Receiving payment for being on a panel or for writing a brief does NOT constitute employment. Both of the times that I received a payment it was based on a contractual arrangement – I provided a service and received payment.*

a. Please provide an amended Funding Statement declaring this commercial affiliation, as well as a statement regarding the Role of Funders in your study. If the funding organization did not play a role in the study design, data collection and analysis, decision to publish, or preparation of the manuscript and only provided financial support in the form of authors' salaries and/or research materials, please review your statements relating to the author contributions, and ensure you have specifically and accurately indicated the role(s) that these authors had in your study. You can update author roles in the Author Contributions section of the online submission form.

*See my above response. Once again, I was not and am not employed by any commercial entity.* *The study was NOT funded.* *The absence of any funding was already stated when I initially submitted the manuscript.*

Please also include the following statement within your amended Funding Statement.  “The funder provided support in the form of salaries for authors, but did not have any additional role in the study design, data collection and analysis, decision to publish, or preparation of the manuscript. The specific roles of these authors are articulated in the ‘author contributions’ section.” If your commercial affiliation did play a role in your study, please state and explain this role within your updated Funding Statement.

*Please seem my above response. The study was NOT funded. The absence of any funding was already stated when I initially submitted the manuscript.*

b. Please also provide an updated Competing Interests Statement declaring this commercial affiliation along with any other relevant declarations relating to employment, consultancy, patents, products in development, or marketed products, etc.

*Please see my above response. There is no other commercial affiliation to declare beyond what is already contained in the COI statement.*

Within your Competing Interests Statement, please confirm that this commercial affiliation does not alter your adherence to all PLOS ONE policies on sharing data and materials by including the following statement: "This does not alter our adherence to  PLOS ONE policies on sharing data and materials.” (as detailed online in our guide for authors <http://journals.plos.org/plosone/s/competing-interests>) . If this adherence statement is not accurate and  there are restrictions on sharing of data and/or materials, please state these. Please note that we cannot proceed with consideration of your article until this information has been declared.

Please include both an updated Funding Statement and Competing Interests Statement in your cover letter. We will change the online submission form on your behalf.

*Please carefully read what I have written above. The study was NOT funded. I was not and am NOT employed by any commercial entity.*

3. Please review your reference list to ensure that it is complete and correct. If you have cited papers that have been retracted, please include the rationale for doing so in the manuscript text, or remove these references and replace them with relevant current references. Any changes to the reference list should be mentioned in the rebuttal letter that accompanies your revised manuscript. If you need to cite a retracted article, indicate the article’s retracted status in the References list and also include a citation and full reference for the retraction notice.

*The reference list has been reviewed and it is complete and correct.*

**Response to Reviewer 1**

I very much enjoyed the paper. It is a simple - but far from simplistic - comparison of drug approvals, using Prescrire as the accepted external standard. It answers a worthy regulatory question - what is being approved and if indications are sound - even if the 'why' cannot be ascertained. This work has the potential for application in multiple contexts. It also makes an important case against the sometimes perilous application of reliance - perhaps not the intention of the paper, but certainly enhances the point that independent analyses are something regulators should not discard. Two issues to consider:

*I appreciate the support from the reviewer for this article.*

1) The meaning of the term 'minor value' (Discussion Section, page 15, line 5) is not clear. Please explain or maybe amend;

*“Minor value” has been replaced by the phrase “only slight or no improvements over existing drugs as rated by the PMPRB”.*

2) Please consider adding the material in the Supplement to the core text. The table makes all the difference and is a crucial element for readers to fully understand the depth of this analysis. Without it numbers and proportions do not really get to the point. Readers must see the drugs and indications.

*The Supplementary Table is very large. If the editors request that it be included as part of the main article I would be happy to do so, but in the meantime I have added a table that gives the generic name and indications of the 99 drug-indications rated by Prescrire as not acceptable.*

In closing, Prescrire´s much more holistic outlook - in contrast to the isolated view of RAs - is what we aim for - and consistent with appropriateness of drug use and with the EM concept, which drug approval processes worldwide must go back to, if only for the sake of equity and sustainability, if not for upholding adequate standards of care.

**Response to Reviewer 2**

This study aims to assess the status of drugs in Canada that have been rated as ‘not acceptable’ by the French drug bulletin Prescrire. In general, the manuscript adheres to most of PLOS ONE’s publication criteria. However, especially the analyses performed and the reporting and contextualization of the results require some more depth. Therefore, I deem several major revisions necessary before I can recommend it for publication. In that light, I have several questions and suggestions for the author to clarify the study and improve the analyses and the presentation of the results.

**Major**

- Although highly specialised readers in this field may instantly appreciate the choice of using Prescrire’s ratings of drugs, the manuscript lacks a clear rationale why this study aims to use data from Prescrire for the Canadian context. The introduction scarcely mentions what Prescrire is.

*I have added a brief description of Prescrire along with a reference in the first paragraph in the Introduction as well as an explanation of why Prescrire ratings are being used: “**Although bodies such as the French* *Haute Autorité de Santé and the Institute for Quality and Efficiency in Health Care, the German health technology assessment agency, evaluate the therapeutic value of new drugs using an ordinal scale and publish their findings in English, the evaluations from Prescrire are used in this study because it is the only organization that names drugs that should not have been given regulatory approval.”*

- Similarly, the Limitations section (p.11) states that there is no independent confirmation of Prescrire’s ratings. However, several studies have compared therapeutic value as assessed by Prescrire with that assessed by (European) HTA agencies. Their findings add important context and shed light on the generalisability of Prescrire’s ratings. I thus recommend the author to clarify the choice of using Prescrire to make the study more accessible and understandable for non-specialists as well as to readers who are familiar with assessing the therapeutic value of new drugs.

*Prescrire’s ratings have been compared to those from the bulletin of the Swedish Medical Products Agency and from the French regulatory agency Haute Autorité de Santé and I have cited this literature. I have also modified the statement in the Limitations section.*

- While a lot of studies have evaluated therapeutic value of new drugs as assessed by (different) regulatory authorities and differences between them and HTA organisations, the manuscript lacks grounding in and connection of its findings and rationale to these studies and discussions. I believe this would add to the manuscript’s relevance and rationale when these discussions are referred to and reflected upon both in the introduction and the discussion section.

*Prescrire’s ratings are compared to those produced by the Patented Medicine Prices Review Board (PMPRB), but the PMPRB is not a health technology assessment body. (That function is assumed by the Canadian Agency for Drugs and Technologies in Health.) The PMPRB assesses the additional therapeutic value of new patented drugs compared to drugs already on the Canadian market and that assessment is used in the process of setting a maximum introductory price for new patented drugs. Therefore, I have not discussed the relationship between the regulatory approvals made by Health Canada and HTA recommendations, but I have made the role of the PMPRB clear.*

- The study’s Methods are in principle fit for answering the presented research question, yet some explanation would help to understand why and which steps were undertaken. For instance, it is yet unclear why data about the completion of the post-authorisation conditions were collected for drugs with a NOC/c approval and why the safety advisories were collected. Moreover, providing more context and detail to the data analysis section would help in understanding why steps are taken.

*Information about the type of approval given by Health Canada has now been removed from the Methods and Results sections. The Methods section contains an explanation about why information was collected about safety reports and the PMPRB therapeutic evaluations: “An attempt was made to produce a Canadian assessment of the benefit to harm ratio of not acceptable drugs on the Canadian market to evaluate how these assessments compared to the ones from Prescrire. Accordingly, the Recall and Safety Alerts website was searched for safety advisories issued by Health Canada for drugs in this group [11] accompanied by a search of therapeutic evaluations done by the Patented Medicine Prices Review Board (PMPRB) and published in its annual reports [12]. The PMPRB is an independent quasi-judicial body that reviews the prices of new patented medicines sold in Canada and as part of that function undertakes therapeutic evaluations and categorizes the drugs as breakthrough, substantial improvement, moderate improvement, slight or no improvement. (The PMPRB does not evaluate the therapeutic value of new indications for existing drugs.)”*

*The Methods section also now explains why the difference in approval dates was calculated between the EMA and Health Canada: “Prescrire evaluations and Health Canada approvals may have differed due to the two organizations being presented with different information. Prescrire evaluations are undertaken shortly after EMA approval and therefore, the difference between the EMA and Health Canada approval dates (in days) was calculated and the median and interquartile range was reported to explore this possibility.”*

- In relation, the completion status and safety advisories not necessarily only lead to drug withdrawals, although the last sentence of the first Results paragraph (p.8, first sentence) and the second-last Results paragraph do suggest this. This should be further explained.

*The last sentence in the first paragraph in the Results section was not intended to mean that drugs with safety issues are always withdrawn from the market. The sentence was part of the description about the EMA regulatory status of the not acceptable drugs. The second last Results paragraph has been deleted.*

- In general, the Results section requires restructuring and additional headings to better guide the reader through the study’s findings and their relevance.

*Additional subheadings have been added in the Results section. The subsection in the Results* *“Not acceptable drugs approved by Health Canada” has been restructured to make it easier to follow.*

- To support and make the study’s findings more intelligible, the Results section would benefit from adding a table and/or figure (such as bar or pie charts) in which the findings are presented. Similarly, a typical ‘Table 1’ would provide required information on the drugs and indications included in the study (n=62/70). Figure 1 would benefit from a clearer visual distinction between the included and excluded drugs. Of note, the ‘safety advisories issues’ now suggest exclusion of these drugs from the cohort. Moreover, clarifying how the results and percentages relate to the different ‘buckets’ of medicines would be helpful for readers.

*Table 1 giving the generic names and indications of the not acceptable drugs approved by the EMA and the French regulatory authority has been added. Figure 1 has been replaced with a new bar chart that also clearly indicates the reasons why drugs were included and excluded.*

**Minor**

- I recommend to revise the title and abstract to clarify that the ‘not acceptable’ rating is as considered by Prescrire. Now, they suggest that the study assessed drugs that were refused for marketing authorization by Health Canada. This should also be clarified throughout the manuscript.

*The title has been changed and now reads: “Were drug-indications evaluated by Prescrire as “not acceptable” approved by Health Canada: a cohort study”. The manuscript makes it clear that a minority of these drugs were refused market authorization by Health Canada.*

- As mentioned in the Limitations section, the data collection has been performed only by the author himself. Given the vast number of bulletins that needed to be checked manually, I would like to emphasise the importance to validate at least a sample by a second researcher.

*I appreciate the concern of the reviewer. In the absence of a second researcher, I have gone through the issues of Prescrire again to verify that all of the drugs rated as not acceptable have been included.*

- I must admit that I could not locate the difference in appraisals between Prescrire and Health Canada as discussed in the second sentence of the abstract, in the Results section. At least, it was not explicitly discussed.

*Health Canada does not offer an appraisal of the therapeutic value of new drugs, it either approves them for marketing or rejects them. (At times, the manufacturer withdraws the submission before Health Canada has reached a decision.) The Canadian status of the 99 drug-indications is described in the Results subsection* *“Not acceptable drugs approved by Health Canada”.*

- The last paragraph of the Results (“Twenty-seven of the (…) in favour of the EMA/ANSM” (p.9) may be moved more forward, for a more logical flow of the Results section.

*The paragraph in question deals with the difference in the time between EMA and Health Canada approvals. Changing its position would result in its placement before the results about how many drug-indications were approved by Health Canada and would lead to confusion. Therefore, I have left the paragraph where it is.*

- The manuscript’s intelligibility would profoundly benefit from more detail and explanation throughout the manuscript, including:
- Explain the relation between the therapeutic evaluations by the PMPRD and the study’s objective (as for instance mentioned in abstract)

*The Methods section now explains the reason for examining the therapeutic assessments by the PMPRB.*

- How are ‘drug-indications’ defined?

*A definition of “drug-indication” has been added to the end of the first paragraph in the Introduction.*

- The methods section describing the data analysis is rather concise, yet, more clarification would help the reader to understand how this relates to the results presented

*The data analysis section now explains the rationale for calculating the time difference between the EMA and Health Canada approvals.*

- Introduction:
  - First paragraph, the ‘EMA’s decentralized procedure’ is not governed by EMA nor does it allow approval in a single market per definition

*I’m afraid that I do not follow the reviewer’s comment. The following quote comes from the EMA webpage (https://www.ema.europa.eu/en/glossary/decentralised-procedure) describing the decentralized procedure: “The procedure for authorising medicines in more than one European Union Member State in parallel. It can be used for medicines that do not need to be authorised via the centralised procedure and have not already been authorised in any Member State.” If the reviewer can be more specific, I would be happy to make any necessary corrections.*

- Clarify the objective: is the aim to assess only whether they were approved? Should be aligned.

*As stated at the end of the Introduction, the objective of the study was to determine if the not acceptable drugs were approved by Health Canada. I am not sure what the reviewer is referring to when he says “should be aligned” but I have changed the final sentence in the Introduction in the Abstract to conform to the one that is at the end of in the Introduction in the main text.*

- Methods/Results: why is the formulation relevant for the objective? If necessary, please explain.

*The formulation of drugs may affect their ease of use, how quickly they are absorbed and how often they need to be used. Differences in these characteristics may contribute to different assessments of whether drugs should be on the market.*

- The last sentence of second paragraph of the Discussion on p10 is a relevant observation. Could such differences in evaluation outcomes between PMPRB and Prescrire be due to differences in availability of alternative treatments in Canada vs. France/the EU?

*The reviewer is correct, drugs may be rejected in one jurisdiction because better treatments are available and accepted in another jurisdiction because these alternatives are not available. However, comparing alternatives in Canada and France for 99 different drug-indications would be a very large undertaking and beyond the resources that I have available.*

- Also, given the highly related topic of ref 12, it should also be discussed in the introduction.

*I agree that the topics are related but I am not clear why the reviewer feels that this relationship requires a discussion of reference 12 in the Introduction. I would appreciate a bit more clarity.*

- The Data Availability Statement states that all data are available in supplementary, however, is the data from Prescrire approved to be made available?

*I have emailed Prescrire requesting permission to use its reasoning about why the drugs were assessed not acceptable.*

- Finally, the manuscript would be improved in clarity and coherence by specifying the actors in several sentences. For instance, in the sentences below Figure 1 on page 8-9: who rated the ‘slight/no improvement’; and who did the ‘therapeutic evaluations’; which review process. Also in the second sentence of the Discussion: who performed the therapeutic ratings. Etc. etc.

*The sentence below Figure 1 now makes it clear that the PMPRB did the ratings. Further on in the paragraph it now states that the therapeutic evaluations were done by the PMPRB. The second sentence in the Discussion also specifies that the therapeutic ratings were done by the PMPRB.*
