# Supplementary material for: Canadian status of “not acceptable” drugs as evaluated by Prescrire: A cohort study
Source: PLoS One. 2024 Aug 1;19(8):e0308118. doi: 10.1371/journal.pone.0308118 (PMC11293654; doi:10.1371/journal.pone.0308118)
Supplement: Attachment — Submitted filename: 20240620-PONE-D-24-11119_R1-review_comments.docx [file pone.0308118.s004.docx]

**Title: Were drug-indications evaluated by Prescrire as "not acceptable" approved by HealthCanada: a cohort study**

**Journal: PLOS ONE**

**Manuscript number: PONE-D-24-11119**

Reviewers’ Comments:

Dear Author, dear Editors,

I thank the author for the response to the comments I made on the previous version of the manuscript. Several of the issues have been adequately addressed and resolved. However, based on the responses, I deem several revisions still necessary before I can recommend it for publication. I have several outstanding questions and suggestions for the author to clarify the study and improve the analyses and the presentation of the results.

**Major**

- I strongly recommend to better ground the manuscript in and connect its rationale and findings to the larger discussion in the scientific literature about the discrepancies between regulatory approvals (e.g., by the EMA or Health Canada) and the added value for society of new drugs. Prescrire is not the only organisation that assesses the therapeutic/clinical/added(/societal?) value of new drugs, but a discussion of, for example, discrepancies between regulatory and HTA evaluations or the role of other value scales is lacking. This is crucial to understand the broader context of the manuscript and to strengthen its rationale.
- An explanation is required in the introduction and/or the methods section to understand why the author chose to use the PMPRB ratings and not those of the Canadian Agency for Drugs and Technologies in Health (CADTH), as well as how their position and evaluations relate to each other.
- The author now clarified that he aimed to simulate a ‘Canadian benefit to harm ratio’ by combining PMPRB ratings and whether safety advisories were issued. This was not clear from the initial manuscript. However, it begs the question what the safety advisories add to this assessment because:

1. Doesn’t PMPRB already consider safety aspects in their evaluation of therapeutic improvement? If they do, please explain the added value of the safety advisories to the consideration of safety by the PMPRB.
2. My understanding of the safety advisories is that they are distributed post-approval by Health Canada. However, the drug-indication combinations in the cohort did not have similar follow-up and thus not the same risk time to receive a safety advisory. If correct, this could substantially bias the analysis.
3. In addition, the author specifically highlights for the drug-indication combinations for which safety advisories were issued, the proportion for which a PMPRB evaluation was available. This also biases the analysis and should be the other way around: n safety advisories/n PMPRB evaluations.

Please correct me if the above is incorrect and explain why. If not, please revise the manuscript to address these issues.

- Page 13 states that “1 drug had been removed from the market for safety reasons”. The manuscript currently suggests that this drug was excluded from the analysis, while, if ever approved by Health Canada, it should have been included and carried forward for analysis in the section about “Canadian evaluations”. Please revise the manuscript and Figure 1 accordingly.
- I would like to urge the author to adapt the newly added and very lengthy Table to a more general ‘Table 1’ with some aggregated characteristics to describe the cohort. For instance by including the following elements from the supplementary material: disease areas, period of approval and whether it was initially approved by EMA and/or ASNM, ND/NI.
- Additionally, a flowchart that visualises which subset of drug-indications are used for the several analyses throughout the manuscript would substantially increase the intelligibility of the manuscript.

**Minor**

- The study’s objective is not solely about the approval by Health Canada but the title does suggest this. Please change the title to bring it more in line with the study’s objective and broader scope, for instance to read: “Status of not acceptable drugs as considered by Prescrire in Canada: a cohort study”.
- The manuscript’s intelligibility would profoundly benefit from more detail and explanation throughout the manuscript. Please consider the following points:
- Mention in the introduction that previous research has shown that drugs that should be avoided according to Prescrire are in fact available in Canada. This adds to the rationale and context for the study too. (about the reference Lexchin *et al*., 2017)
- Introduction: the wording of ‘by the EMA’s decentralised procedure … a single market’ is not correct here, please adapt: “approved for marketing in France either by ASNM or the EMA’.
- The author performs no assessment of the ‘benefit to harm ratio’ (like the EMA or Health Canada assess the benefit-risk), but he performs rather a ‘Canadian assessment of the added/clinical/therapeutic value’. Please clarify this accordingly throughout the manuscript.
- The Data Analysis section should be revised to clarify which analyses were performed and how.
- In the Results section ‘Not acceptable drug approved by Health Canada’, adding that the 99 drug-indications concern those that were deemed ‘not acceptable’ by Prescrire would add clarity about the cohort characteristics
- The relevance for extracting and reporting the drug formulation is made clear in the author’s answer to the question. Please add this reasoning to the methods section, as well as the fact that the formulation was extracted for each drug, in addition to the generic name, brand name, etc.
