# Supplementary material for: Canadian status of “not acceptable” drugs as evaluated by Prescrire: A cohort study
Source: PLoS One. 2024 Aug 1;19(8):e0308118. doi: 10.1371/journal.pone.0308118 (PMC11293654; doi:10.1371/journal.pone.0308118)
Supplement: Attachment — Submitted filename: Response to reviewers.docx [file pone.0308118.s005.docx]

I am grateful to the reviewers for the time that they have taken to examine the manuscript.

**Reviewer #1**

Table 1 was a great gain to the paper.
Clarification of the term 'minor value' was resolved with substitution.
I did not encounter the difficulties presented by Reviewer 2, but no harm was done with the answers to the multiple queries.
The manuscript is sound and well-presented.

*I appreciate the reviewer’s confidence in the quality of the article.*

**Reviewer #2**

**Major**

- I strongly recommend to better ground the manuscript in and connect its rationale and findings to the larger discussion in the scientific literature about the discrepancies between regulatory approvals (e.g., by the EMA or Health Canada) and the added value for society of new drugs. Prescrire is not the only organisation that assesses the therapeutic/clinical/added(/societal?) value of new drugs, but a discussion of, for example, discrepancies between regulatory and HTA evaluations or the role of other value scales is lacking. This is crucial to understand the broader context of the manuscript and to strengthen its rationale.

*I appreciate the reviewer’s point about understanding the overall value of drugs to society, but that is not the goal of this manuscript. The primary goal is to examine whether Health Canada has approved drugs that Prescrire has determined should not have been approved by drug regulatory agencies. Importantly, drug regulatory agencies and health technology agencies (HTAs) serve two distinct functions. Drug regulatory agencies evaluate the results of pivotal trials to decide on the efficacy of the new product (i.e., is the drug superior to placebo) and is it safe enough to be used for its intended indication. HTAs evaluate whether a drug delivers value for the price and depending on the indication the cost per QALY may be variable. Therefore, a drug that the regulatory agency approves may be rejected for funding by agency performing the HTA because of low efficacy (but still superior to a placebo), high price or a combination of both. I would like the academic editor to take a position about whether I should follow through with the request from the reviewer.*

- An explanation is required in the introduction and/or the methods section to understand why the author chose to use the PMPRB ratings and not those of the Canadian Agency for Drugs and Technologies in Health (CADTH), as well as how their position and evaluations relate to each other.

*The PMPRB evaluates the additional therapeutic value of new drugs. CADTH makes recommendations about whether new drugs for particular indications should be funded. These are two different types of activities. CADTH might recommend reimbursement for a drug that is no better or worse than existing products as long as its price is reasonable or recommend not funding a drug not because the drug has a negative benefit to harm ratio but because the drug does not represent value for money. CADTH’s recommendations about funding cannot be directly translated into a categorical rating of the additional therapeutic value of drugs. Since the manuscript does not mention CADTH I do not feel the need to discuss its role in the context of drug funding in Canada.*

- The author now clarified that he aimed to simulate a ‘Canadian benefit to harm ratio’ by combining PMPRB ratings and whether safety advisories were issued. This was not clear from the initial manuscript. However, it begs the question what the safety advisories add to this assessment because:

1. Doesn’t PMPRB already consider safety aspects in their evaluation of therapeutic improvement? If they do, please explain the added value of the safety advisories to the consideration of safety by the PMPRB.

*The PMPRB considers the efficacy and safety of new drugs largely based on the documentation submitted by companies at the time when they apply for approval. Safety advisories are typically based on new information about issues that arise post-marketing and would not have been identified in the material submitted for approval.*

2. My understanding of the safety advisories is that they are distributed post-approval by Health Canada. However, the drug-indication combinations in the cohort did not have similar follow-up and thus not the same risk time to receive a safety advisory. If correct, this could substantially bias the analysis.

*The reviewer is correct that safety advisories are issued post-approval based on newly identified risks associated with the drug. As stated in the Methods section, a search was made for any post-market safety advisories issued for all the drug-indications approved by Health Canada. Therefore, all the drug-indications approved by Health Canada had the same follow-up in terms of looking for new safety issues.*

3. In addition, the author specifically highlights for the drug-indication combinations for which safety advisories were issued, the proportion for which a PMPRB evaluation was available. This also biases the analysis and should be the other way around: n safety advisories/n PMPRB evaluations.

*I am unclear why the reviewer thinks that what I have said about the number of drugs with PMPRB evaluations and the number with safety evaluations biases the analysis. I believe that the revised final sentence on page 13 is clear: “Therefore, a separate Canadian assessment of the therapeutic value and safety was only possible for a small minority of not acceptable drugs, i.e., 5 of the 70 drug-indications were slight to no improvements and had serious safety issues.”*

Please correct me if the above is incorrect and explain why. If not, please revise the manuscript to address these issues.

- Page 13 states that “1 drug had been removed from the market for safety reasons”. The manuscript currently suggests that this drug was excluded from the analysis, while, if ever approved by Health Canada, it should have been included and carried forward for analysis in the section about “Canadian evaluations”. Please revise the manuscript and Figure 1 accordingly.

*Presumably the reviewer is referring to fenfluramine. The text and Figure 1 have been revised to state that the drug was removed from the market and never submitted to Health Canada for approval.*

- I would like to urge the author to adapt the newly added and very lengthy Table to a more general ‘Table 1’ with some aggregated characteristics to describe the cohort. For instance by including the following elements from the supplementary material: disease areas, period of approval and whether it was initially approved by EMA and/or ASNM, ND/NI.

*Adapting the table in the manner requested by the reviewer would create a very large table. Making this change could easily be done, but I would appreciate guidance from the academic editor regarding whether he agrees with this request.*

- Additionally, a flowchart that visualises which subset of drug-indications are used for the several analyses throughout the manuscript would substantially increase the intelligibility of the manuscript.

*Figure 1 provides information and a count about the number of drugs approved by Health Canada and the reasons why drug-indications were excluded. There are only two other analyses undertaken. One is a count of the number of drugs with PMPRB evaluations and safety warnings and the second is the difference in approval times between Health Canada and the EMA. I don’t believe that a flowchart is necessary to be able to follow these analyses, but once again would appreciate guidance from the academic editor.*

**Minor**

- The study’s objective is not solely about the approval by Health Canada but the title does suggest this. Please change the title to bring it more in line with the study’s objective and broader scope, for instance to read: “Status of not acceptable drugs as considered by Prescrire in Canada: a cohort study”.

*The title has been changed to “Canadian status of “not acceptable” drugs as evaluated by Prescrire: a cohort study”.*

- The manuscript’s intelligibility would profoundly benefit from more detail and explanation throughout the manuscript. Please consider the following points:
- Mention in the introduction that previous research has shown that drugs that should be avoided according to Prescrire are in fact available in Canada. This adds to the rationale and context for the study too. (about the reference Lexchin *et al*., 2017)

*The change suggested by the reviewer has been made near the end of the Introduction.*

- Introduction: the wording of ‘by the EMA’s decentralised procedure … a single market’ is not correct here, please adapt: “approved for marketing in France either by ASNM or the EMA’.

*The end of the paragraph now reads “The date of approval of the drug-indications marketed in France was found by searching on the drug’s name on either the EMA website [7] or the website of the French A**gence nationale de séurité du medicament et des produits de santé (ANSM) [8].”*

- The author performs no assessment of the ‘benefit to harm ratio’ (like the EMA or Health Canada assess the benefit-risk), but he performs rather a ‘Canadian assessment of the added/clinical/therapeutic value’. Please clarify this accordingly throughout the manuscript.

*The reviewer is not correct. I do not attempt to perform “a Canadian assessment of the added/clinical/therapeutic value”. What I have done is to look at additional therapeutic value as assessed by the PMPRB and safety based on safety advisories. However, I have changed the opening sentence of the paragraph on page 6 to read “An assessment was made of the additional therapeutic value and the safety of not acceptable drugs on the Canadian market.” A similar change was made in the “Canadian evaluations of the therapeutic benefits and harms of not acceptable drug-indications” subsection of the Results.*

- The Data Analysis section should be revised to clarify which analyses were performed and how.

*There are only three analyses that are done. The first is a count of the number of not acceptable drugs that Health Canada has approved. The second are counts of the number of drugs that have been evaluated by the PMPRB and that have safety warnings and the third is a calculation of the difference in the time between when the EMA and Health Canada approved the drugs. The first and third analyses are already described in the Data Analysis section. I have now added that counts were made of the number of drugs with PMPRB evaluations and with safety warnings.*

- In the Results section ‘Not acceptable drug approved by Health Canada’, adding that the 99 drug-indications concern those that were deemed ‘not acceptable’ by Prescrire would add clarity about the cohort characteristics.

*The first sentence in this section now reads “Out of the 99 drug-indications identified by Prescrire as not acceptable…”*

- The relevance for extracting and reporting the drug formulation is made clear in the author’s answer to the question. Please add this reasoning to the methods section, as well as the fact that the formulation was extracted for each drug, in addition to the generic name, brand name, etc.

*The information about why the formulation of the drug was recorded has been added to the Methods section.*
